# Effect of beetroot juice on lowering blood pressure in free-living, disease-free adults: a randomized, placebo-controlled trial

**DOI:** 10.1186/1475-2891-11-106

**Published:** 2012-12-11

**Authors:** Leah T Coles, Peter M Clifton

**Affiliations:** 1 Nutritional Interventions Laboratory (LC, PC), Baker IDI Heart & Diabetes Institute, Melbourne, Australia

**Keywords:** Beetroot, Blood pressure, Hypertension, Inorganic nitrate

## Abstract

**Background:**

The consumption of beetroot juice on a low nitrate diet may lower blood pressure (BP) and therefore reduce the risk of cardiovascular events. However, it is unknown if its inclusion as part of a normal diet has a similar effect on BP. The aim of the study was to conduct a randomized controlled trial with free-living adults to investigate if consuming beetroot juice in addition to a normal diet produces a measureable reduction in BP.

**Method:**

Fifteen women and fifteen men participated in a double-blind, randomized, placebo-controlled, crossover study. Volunteers were randomized to receive 500 g of beetroot and apple juice (BJ) or a placebo juice (PL). Volunteers had BP measured at baseline and at least hourly for 24-h following juice consumption using an ambulatory blood pressure monitor (ABPM). Volunteers remained at the clinic for 1-h before resuming normal non-strenuous daily activities. The identical procedure was repeated 2-wk later with the drink (BJ or PL) not consumed on the first visit.

**Results:**

Overall, there was a trend (*P*=0.064) to lower systolic blood pressure (SBP) at 6-h after drinking BJ relative to PL. Analysis in men only (*n*=13) after adjustment for baseline differences demonstrated a significant (*P*<0.05) reduction in SBP of 4 – 5 mmHg at 6-h after drinking BJ.

**Conclusions:**

Beetroot juice will lower BP in men when consumed as part of a normal diet in free-living healthy adults.

**Trial registration:**

anzctr.org.au ACTRN12612000445875

## Background

In the last 20 years or so there has been renewed interest in the potential of inorganic nitrate (NO_3_^-^) to control blood pressure in humans [1]. Dietary inorganic nitrate is absorbed rapidly and completely in the proximal small intestine with 100% bioavailability [2]. Approximately 25% of the nitrate circulating in the plasma is then concentrated in the salivary glands and secreted into the mouth where around 20% (or ≈ 5 – 8% of intake) is converted to nitrite (NO_2_^-^) by commensal bacteria on the tongue and subsequently swallowed [3]. Upon reaching the stomach the NO_2_^-^ is either absorbed directly or reduced to nitric oxide (NO) as a result of the acidic environment of the stomach [3-5]. Endogenously produced NO and NO_2_^-^ are vasoprotective agents with the ability to increase vasodilation, decrease blood pressure (BP) and improve cardiovascular function [6]. Reduced endogenous NO production is associated with hypertension [7] and there is evidence to support the hypothesis that the NO and NO_2_^-^ produced as a result of dietary NO_3_^-^ could induce health benefits [8].

The individual daily intake of dietary nitrate has been estimated to be ≈81–106 mg/d (not including loses from washing, peeling and cooking) in the typical Western diet, with vegetables contributing approximately 80% of this value [9,10]. The vegetables with the highest nitrate contents (>250 mg/ 100 g fresh weight) are celery, cress, chervil, lettuce, red beetroot, spinach and rocket [11]. Green leafy vegetables have recently been shown to be among the foods most beneficial in the prevention of coronary heart disease and ischemic stroke [12,13]. This effect has been postulated to be due to the high inorganic NO_3_^-^ content of these vegetables [14-16].

Beetroot is a rich source of dietary NO_3_^-^[11] and a number of studies have investigated its potential for reducing blood pressure in humans [8,17-20], which appears to be more potent in men.

Although beetroot appears to be a promising means of lowering blood pressure, previous studies in free-living subjects have been small in size and routinely used a controlled diet in combination with administering the beetroot juice. It is not clear whether a blood pressure-lowering effect may be seen when beetroot juice is consumed on top of a normal diet. The present study examines the use of beetroot juice as a means of regulating blood pressure in free-living individuals whilst not imposing any dietary restrictions.

## Methods

### Materials

The nitrate was administered as Sunraysia Beetroot and Apple Juice (72% beetroots and 28% apples) (BJ), containing 15 mmol nitrate/L (data supplied by The Sunraysia Natural Beverage Company, Melbourne, VIC). The placebo (PL) consisted of an apple juice concentrate base matched to the BJ for sweetness (sucrose) and colour (carmine red and rubini red). Both the BJ and PL were supplied by The Sunraysia Natural Beverage Company, Melbourne, VIC.

### Intervention study subjects

A total of thirty healthy volunteers (15 F, 15 M) were recruited for the trial from Melbourne, Australia. The primary inclusion criterion was a systolic blood pressure (SBP) of greater than 120 mmHg at the time of administering the first treatment. Subjects who were pregnant/lactating, on blood pressure medication or had been diagnosed with diabetes mellitus were excluded from participating. Written informed consent was obtained from all volunteers prior to participation in the study. The study was approved by the Alfred Research Human Ethics Committee (377/11) and took place in January 2012.

### Study design

The study was designed as a double-blind, randomized, crossover, intervention trial in which volunteers were asked to consume 500 g of either BJ or PL on a single occasion. No dietary restrictions were placed on the subjects prior to visits, in the 24 h period following consumption of the BJ or PL or during the 2-wk washout period.

Subjects arrived at the clinic at approximately 0800 h on the day of treatment in a non-fasted state (light breakfast only), having performed no more than light physical activity on the study day. Subsequent to anthropometric measurements (height and body weight) and a rest period of 5-min, subjects were fitted with an ABPM (Meditech ABM-04, Budapest, Hungary) to measure SBP, diastolic blood pressure (DBP) and pulse rate. Three baseline blood pressure measurements were recorded by the ABPM at intervals of 2-min apart with the subject in a seated position. If the subject recorded an average baseline SBP of greater than 120 mmHg they were randomly assigned by computer software to receive the BJ or PL, which was then consumed immediately in unmarked glasses over the course of 5 to 10-min. Subjects remained in a seated and unfed state at the clinic for 1-h post-drink consumption, during which time three BP measurements were taken by the ABPM. Subjects then left the clinic and resumed normal daily tasks whilst continuing to wear the ABPM for a further 23-h. Subjects were instructed not to undertake any moderate or intense physical activity (e.g. brisk walking, cycling, running, gym workout) during this time and to loosely hang their cuffed arm by their side during BP readings. ABPM measurements were automatically taken half-hourly during the active period (0700 h – 2300 h) and hourly during the remaining passive period. An activity log book was also given to volunteers to record actual awake and sleeping times and the times of any ‘unusual’ events that may have effected BP measurements (e.g. getting a parking ticket, running late for a bus). Subjects returned to the clinic 24-h following the treatment to have the ABPM removed. Subjects returned to the clinic 2-w later, and repeated the same methodology with the drink (BJ or PL) not consumed on the first visit.

### Statistical analyses

Power calculations were based on a previous similar study [8] where the SD of the change in BP at 2.5-h was 6 mmHg. With 30 participants completing the present study using a crossover design it was calculated that a 3.2 mmHg difference (or 4.7 mmHg difference in men alone) in BP could be detected with 80% power (*P*<0.05).

The data from the BP monitors was transferred into Microsoft Excel (Microsoft Corp., Redmond, WA) and then analyzed using statistical software (SPSS 18, IBM Corp., Armonk, NY). The ABPM provided all raw data as well as averages for active/ day time (0600 – 2259 h) or passive / night time (2300 to 0559 h) periods. Averages were calculated for baseline BP from the 3 measures taken, over 3 to 4-h, 6 to 7-h or 23 to 24-h after the drink. Depending on the number of successfully recorded measurements in each period, there may have been 1–3 values in each of these time periods. Repeated measures analysis of covariance was used with a covariate of the difference between baseline blood pressure on the two days (ANCOVA). A separate analysis with a post hoc removal of outliers (>3SD drop in blood pressure after ingestion of the juice) was also performed. Given the previous results showing men showed greater effects, a planned analysis in men only was performed. In all cases, differences were deemed significant at *P*<0.05.

## Results

Satisfactory blood pressure recordings were reported by the 15 men and 15 women (Table 1) who completed the study and no adverse effects were reported from drinking the intervention juices. Subjects ranged in age from 23 – 68 y. Subjects were generally healthy with none of the male subjects and approximately half of the female subjects not medicated (Table 2). The number of measures of blood pressure over the 24-h ranged from 31 to from 44 measurements.

**Table 1:** **Baseline characteristics of study subjects**^**1**,**2**^

	**Subjects**					
	**All (*n***=**30)**		**Male (*n***=**15)**		**Female (*n***=**15)**	
**Characteristic**	**Mean**	**SEM**	**Mean**	**SEM**	**Mean**	**SEM**
Age (y)^1^	42.5	3.4	36.2	2.9	48.9	3.1
BMI (kg/m^2^)^1^	28.2	1.3	28.7	1.3	27.6	1.2
SBP (mmHg)^2^	132.4	1.6	132.1	2.2	132.7	2.3
DBP (mmHg)^2^	81.1	1.2	79.6	1.8	82.6	1.7

^1^ Values for Age and BMI are means and their SEM on day of first treatment.

^2^ Values for SBP and DBP are pooled means of the average baseline measures across the two treatment days and their pooled SEM.

BMI, body mass index; DBP, diastolic blood pressure; SBP, systolic blood pressure.

**Table 2:** **Number of study subjects normally taking medication** / **nutritional supplements**, **exercising regularly**, **drinking alcohol and smoking cigarettes**

	**Number of subjects partaking**		
**Medication** / **Supplement** / **Lifestyle factor**	**All** (***n***=**30**)	**Male** (***n***=**15**)	**Female** (***n***=**15**)
Exercise	22	12	10
Alcohol	22	12	10
Cigarettes	3	2	1
Vitamins / fish oil	14	6	8
No prescription medication	22	15	7
Prescription medication	8	0	8
Oral contraceptives / estrogens	5	-	5
Cholesterol	4	-	4
Antidepressants	2	-	2
Osteoporosis drugs	2	-	2
Asthma drugs	2	-	2

Overall, there was no significant difference in treatments for any parameter except day time pulse pressure (difference between systolic and diastolic pressures) of 1 mmHg higher on the PL treatment (Table 3). There were no statistically significant differences between the juices in average day time or night time blood pressures or heart rate.

**Table 3:** **Systolic blood pressure**, **diastolic blood pressure and pulse pressure of subjects during the active and passive periods in the 24**-**h following ingestion of beetroot juice and placebo juice**^**1**^

	**Mean value over 24**-**h**^**1**^					
	**All subjects** (***n***=**30**)		**Male** (***n***=**15**)		**Female** (***n***=**15**)	
**Measurement**	**Placebo**	**Beetroot**	**Placebo**	**Beetroot**	**Placebo**	**Beetroot**
Active period (awake)						
SBP (mmHg)	128.3 ± 1.9	126.6 ± 1.5	129.6 ± 3.1	128.7 ± 2.3	127.0 ± 2.3	124.6 ± 1.8
DBP (mmHg)	77.8 ± 1.7	77.6 ± 1.3	77.1 ± 2.5	77.5 ± 2.0	78.4 ± 2.3	77.9 ± 1.8
Pulse pressure (mmHg)^2^	50.6 ± 1.3^a^	49.1 ± 1.3^b^	52.5 ± 2.1	51.2 ± 2.0	48.7 ± 1.6	46.9 ± 1.4
Passive period (asleep)						
SBP (mmHg)	111.0 ± 2.2	113.3 ± 2.0	111.6 ± 2.6	117.0 ± 2.9	110.5 ± 3.5	109.5 ± 2.4
DBP (mmHg)	62.8 ± 1.3	65.4 ± 1.3	62.1 ± 2.1	65.9 ± 1.9	63.5 ± 1.6	65.0 ± 1.8
Pulse pressure (mmHg)^2^	48.2 ± 1.5	47.8 ± 1.4	49.5 ± 1.8	51.2 ± 1.9	47.0 ± 2.4	44.5 ± 1.5

^1^ Values are means ± SEM. Values with different superscript letters indicate that differences between beetroot juice and placebo are significant, *P* < 0.05.

^2^ Pulse pressure refers to SBP minus DBP.

DBP, diastolic blood pressure; SBP, systolic blood pressure.

Individual BP changes from baseline after each treatment showed a drop of 4.6 mmHg with BJ and 3.4 mmHg with PL at 3-h, 6.2 mmHg and 2.2 mmHg respectively at 6-h and 4.5 mmHg and 2.3 mmHg respectively, at 24-h. Statistically the 6-h difference was a trend overall (*P*=0.064), with men showing a difference of −4.7 mmHg, (*P*=0.1) and women a difference of −2.5 mmHg (*P*=0.5). In the planned ANCOVA in men only BJ treatment was significantly different from placebo (*P*=0.007) and a treatment covariate interaction was observed (*P*=0.024). In those subjects with little variation in baseline BP measures there was a clear effect of BJ. In those subjects with a large variation in baseline BP measures, SBP did not change with BJ and increased with PL, although the difference between the drinks was smaller.

In a post hoc analysis of outliers, those men with large drops in BP (≥ 20 mmHg SBP) at 6-h following drink consumption on either treatment (*n* =2) were removed. In the remaining 13 men the 6-h difference between PL and BJ of 4.9 mmHg was statistically significant (*P*=0.042).

## Discussion

It has been postulated that the inclusion of dietary nitrates in the form of beetroot-derived foods may be useful in the regulation of normal BP due their high inorganic NO_3_^-^ content. The present contribution is the first study, to the authors’ knowledge, to examine the effect of beetroot juice on BP in free-living individuals in the absence of dietary restrictions, such as a low nitrate diet. Dietary restrictions in studies with beetroot juice and BP remove confounding dietary factors that may have an effect (negative or positive) on BP, thus making interpretation of study results more straightforward. The drawback, however, is that beetroot juice as a nutritional intervention to regulate BP would likely be consumed as part of a normal diet, not as part of a low nitrate diet or in the absence of other dietary components (e.g. coffee, alcohol, black tea) that may affect BP [21-23]. It was therefore uncertain whether there is any clinically relevant benefit from beetroot juice supplementation on BP in the unregulated home environment.

Unsurprisingly, there was a large degree of variation in the BP readings for a given individual both across the two measurement days and throughout each day. This may have been a result of the free-living nature of the study. Whilst there was no significant difference in baseline SBP or DBP between men and women, the trend of BJ lowering BP was stronger in men than in women and a planned separate analysis in men showed that BJ lowered SBP by 4–5 mmHg at 6-h after ingestion. It is uncertain whether these differences between the sexes was a result of gender *per se*, or whether the older age of the women (48.9 ± 3.1 y) compared to the men (36.2 ± 2.9 y) may have influenced the variation seen. It is also notable that approximately half of the women (*n*=8) who participated in the study took prescription medication, whilst none of the men did. The crossover design of the study should have eliminated any individual variation in blood pressure due to any medication taken daily on the two 24 h periods that BP was measured. However, the possibility of the effect of medication or diet (e.g. sodium intake) on the outcomes of the trial cannot be discounted and ideally the experiment should be repeated in non-medicated individuals and the dietary intake recorded. Two of the studies in the literature investigating beetroot juice and BP that used both men and women did not report results by gender [8,20]. The third such study, by Kapil and colleagues tested 24 mmol of KNO_3_ (or KCl) in capsules in a double-blind crossover study in 8 males (baseline BP 126/73 mmHg) and 12 females (baseline BP 102/67 mmHg) and found that the KNO_3_ lowered systolic BP by 9.4 mmHg at 6-h and diastolic by 6 mmHg at 2.5-h (systolic lowering was 6 mmHg at this time point) [19]. Females had no significant fall in blood pressure (3-4/5 mmHg reduction only) despite absolute plasma nitrite rises twice those seen in men. Some of these differences may have been due to the large difference in baseline BP (difference in SBP = 24 mmHg, *P*<0.001) between men and women in that particular study as it was noted that decreases in BP were correlated with baseline BP (*r*= 0.66 to 0.7). In the present study, baseline BP readings were remarkably similar between men (132/80 ± 2.2/1.8 mmHg) and women (133/83 ± 2.3/1.7 mmHg). It is difficult to ascertain if there is in fact a gender-specific response to dietary nitrates.

The drop of 4–5 mmHg observed in the study reported here at 6-h after consumption in men is smaller and with a later peak drop in BP than in other controlled studies with beetroot juice. In an open-label and unblinded study, Webb *et al*. demonstrated that 500 ml of beetroot juice containing 45 mmol/L (or 2.79 g/L) of NO_3_^-^ lowered BP by a maximum of 10/4.8 mmHg at approximately 3-h (p<0.001; n=9 m, 5 f; mean baseline BP: 109/71 mmHg) and the effect persisted in SBP for 24-h [8]. Although it was stated that the effect on BP was related to the increase in plasma nitrite this only accounted for about 7% of the variance. In that study, volunteers were measured seated in clinic and asked to refrain from caffeine-containing drinks or foods with a high nitrate content (green leafy vegetables, beetroot) for 12-h prior to the study and were fasting on the morning of the study.

More recently, Hobbs and workers [18] conducted a single-blind, randomized, controlled, crossover study and observed an almost dose-dependent drop of 20.5/14.6 mmHg at 2-3 h postprandial using beetroot juice containing 5.7 mmol NO_3_^-^ relative to the control (water), and 22.2/18.3 mmHg with twice that dose of NO_3_^-^. Once again, subjects (*n*=18 M, mean baseline BP: 131/82 mmHg) were fasted for 12-h and the diet was restricted (no alcohol or and caffeine) in the 24-h prior to the test and were provided with a standard evening meal for the night before each visit and for the lunch and dinner of the study day. Subjects were also instructed not to take any dietary supplements, vitamins or minerals for 1-wk prior to the study or during the intervention period. Subjects were also required to meet a large number of inclusion criteria including not smoking, not exercising more than 3 times / wk (<20 min/session) and not consuming > 150 ml alcohol / wk. The highly controlled nature of this study may have been responsible for the particularly large decreases in BP observed.

Two other studies have also reported a reduction in BP from beetroot juice, but the number of study participants was small. In an open-label study in 9 volunteers (gender not stated) Kapil *et al*. tested 250 ml beetroot juice (5.5 mmol NO_3_^-^) relative to a water control [19]. A peak reduction in SBP of 5.4 ± 1.5 mmHg was found, whilst diastolic blood pressure changes were not significant. Both plasma NO_3_^-^ and NO_2_^-^ were elevated for 3-h following beetroot juice ingestion. In another study, Vanhatalo *et al*. tested 8 volunteers (*n*=5 M, 3 F; baseline SBP and DBP of 127 ± 6 and 72 ± 5 mmHg, respectively) with 0.5 l beetroot juice daily, in two equal doses in the morning and evening, for 5 days against a placebo (low-calorie blackcurrant juice cordial with negligible NO_3_^-^content) in a randomized, crossover study with a 10-d washout period [20]. On day 5 of the treatment, SBP and DBP were reduced on the beetroot juice by approximately 4% relative to the placebo. In the present study, a single dose of BJ was administered and the effect measured over the next 24-h. It is possible that a cumulative beneficial effect may occur if the BJ was consumed on a daily basis. Further studies are needed to confirm if this is the case and to determine what the optimal daily dose should be in free-living adults. The BJ and PL used here contained 28% apple juice. It is plausible that the apple juice may have had some contrary effect on BP, thus partially negating or cancelling the effect of the beetroot on lowering BP. While the literature actually supports a BP lowering effect of quercetin (found in large amounts in apples) [24], the experiment could be repeated using pure beetroot juice to eliminate any possible effect of other components present in the BJ.

As a higher baseline BP has been correlated with a larger reduction in BP following BJ ingestion [19] it is possible that hypertensive patients could benefit more from beetroot juice consumption than the group of subjects studied here. The volunteers used here were healthy, but unlike a number of other studies, they included both genders, a broad age range (23 – 68 y) and exhibited higher baseline BP values (mean 132/81 ± 1.6/1.2 mmHg). The results presented here are therefore more generalizable to the population as a whole in Western countries, especially given the free-living nature of the study. Using an even larger group of volunteers, with different characteristics (e.g. on average, lower / higher BMI or age) would be further beneficial, as the group studied here were on average middle-aged and slightly overweight. Although it was not undertaken in the present study, it would also be preferable to measure plasma NO_3_ levels before and after consumption of the BJ and PL in order to establish a causative relationship between the NO_3_ present in beetroot and the effect on plasma NO_3_ levels leading to a reduction in BP. This would assist in eliminating other possible causes of the effect seen, such as the diuretic property of beetroot.

## Conclusions

In conclusion, it was demonstrated here that in free-living people consuming an unrestricted diet and a single dose of 500 g of beetroot and apple juice, a trend to lower blood pressure by 4–5 mmHg at 6-h was observed (significant only in men after adjustment for baseline variation). A reduction in SBP in the magnitude of 5 mmHg has been correlated to a cardiovascular mortality reduction of approximately 10% at the population level [25]. Additional studies with beetroot and apple juice in larger groups of free-living men and women are needed to fully assess the efficacy such a dietary intervention at a public health level in the treatment of cardiovascular disease and to determine the exact mechanism of action.

## Abbreviations

ABPM: Ambulatory blood pressure monitor; BJ: Beetroot and apple juice; BP: Blood pressure; DBP: Diastolic blood pressure; PL: Placebo; SBP: Systolic blood pressure.

## Competing interests

The authors declare that they have no competing interests. The Sunraysia Natural Beverage Company (Melbourne, VIC) funded this study but was not involved in its design or the collection, analysis or interpretation of data.

## Authors' contributions

PC designed the study, analysed the data and had primary responsibility for final content. LC conducted the study and drafted the report. Both authors read and approved the final manuscript.
